# Separate and Combined Effects of Supplemental CO_2_, Gibberellic Acid, and Light on Hop Quality and Yield

**DOI:** 10.3390/plants13121670

**Published:** 2024-06-16

**Authors:** William L. Bauerle

**Affiliations:** Department of Horticulture and Landscape Architecture, Colorado State University, Fort Collins, CO 80523, USA; bauerle@colostate.edu

**Keywords:** bracts, CO_2_ enrichment, exogenous gibberellin, flowering crops, lupulin, photoperiod

## Abstract

We investigated the effect of supplemental CO_2_, gibberellic acid (GA_3_), and light on the quality and yield of *Humulus lupulus* L. strobili (cones). When applied separately, CO_2_ and light increased the yield by 22% and 43%, respectively, and had a significant effect on the components of cone mass and quality. Exogenous GA_3_ increased flower set; however, the yield decreased by approximately 33%. Combining CO_2_, GA_3_, and light, and any combination thereof, resulted in significant increases in flower set and cone yield enhancement compared to separate applications. A synergistic effect occurred when some factors were combined. For example, the combination of CO_2_ and light resulted in a yield increase of approximately 122%. The combination of all three resources, CO_2_, GA_3_, and light, resulted in an approximate 185% yield increase per plant. Thus, in comparison to the addition of one supplementary resource, a greater increase in yield resulted from the combination of two or more supplemental resources. Flower set stimulation due to GA_3_ decreased cone alpha- and beta-acid quality attributes, unless combined with CO_2_ and light as additional carbohydrate-generating resources. Additional research is needed to close the hop yield gap between current hop yields and the achievement of the plant’s genetic potential.

## 1. Introduction

The German Reinheitsgebot law of 1516 dictates that beer must be comprised of only four ingredients—barley, hops, water, and yeast. Hops, *Humulus lupulus* L. inflorescences, are pivotal for flavor and aroma in beer. The economic viability of hop production hinges on the dry biomass yield of harvested hops per unit area. Traditional hop breeding efforts, however, have primarily concentrated on disease resistance and the bitterness and flavor components that hops impart in beer. Nevertheless, recent studies indicate that biostimulants and environmental factors can significantly bolster yield [1,2,3].

The number of hops per plant is contingent upon flower set, which can be augmented through exogenous gibberellic acid (GA_3_) application [2,4,5,6,7,8,9]. However, increasing the number of hops per plant may inversely affect individual hop size and quality attributes [6,7,8]. Concentrations of α- and β-acid are the accepted industry standard by which the quality of hop flowers are judged. Therefore, increasing the yield is not of value if it decreases the hop quality. The intricate interplay between flowering and assimilate supply conceivably impacts hop quantity, individual flower size, and overall dry yield per plant [5,6,7,8]. Nonetheless, producing the highest possible yield, while maintaining α- and β-acid quality attributes relative to a genotypes’ potential would increase yield per unit land area.

Studies by Bauerle [1,3] have shown that CO_2_ enrichment enhances hop photosynthesis and assimilate supply, while supplemental light also boosts carbon assimilation [3,10,11]. Conversely, the quantity of hops per plant is influenced by hormonal conditions affecting flower cell divisions during flower set [2]. Previous hormonal flower promotion attempts to increase hop yield generated ambiguous results due to inconsistent GA_3_ application timing and environmental variation among studies [4,5,7,8]. To dissect the effects of carbon supply on hop yield, it is crucial to distinguish the impact from that of flower set. The CO_2_ and light levels used in this study were selected based on their relevance to the photosynthesis saturation point of hop leaves, as identified in previous research [3,10]. Additionally, the selected exogenous GA_3_ concentration and timing of its application were based on studies that have consistently and significantly increased hop cone yield [2,7]. Higher GA_3_ doses, however, affect other hop growth processes such as cell and vegetation expansion [2,7]. The author is unaware of any studies investigating the optimal combinations, quantities, and scheduling of supplemental resources for hops. Therefore, we aimed to investigate the separate and combined effects of supplemental CO_2_, GA_3_, and light on hop cone quality and yield. We hypothesized that inflorescence size is resource-limited, while flower number is influenced by hormonal effects. Consequently, we hypothesized that combining GA_3_ with supplemental CO_2_ and light would increase both flower number and overall dry cone yield per plant without compromising cone quality. The main objectives of this study were to investigate the genetic yield potential for a common commercial hop cultivar (genotype) using combinations of growth-increasing supplemental resources and to estimate the potential gain in hop yield that could be achieved through optimum environmental and phytohormone management practices.

Hop plants’ inherent traits, such as their significant length (>20 m) and expansive size, pose research challenges within confined spaces [12,13,14]. This issue can restrict whole plant replication, impacting experimental design and repetition efforts. Acknowledging the limitations imposed by the characteristics of hop growth, our study focuses on observing hop inflorescence development, growth, quality, and dry cone yield. The experimental variables include supplemental CO_2_, GA_3_, and light, all tested under otherwise uniform environmental conditions.

## 2. Results

### 2.1. Separate Effects of CO_2_, GA_3_, and Light

**Figure 1:**
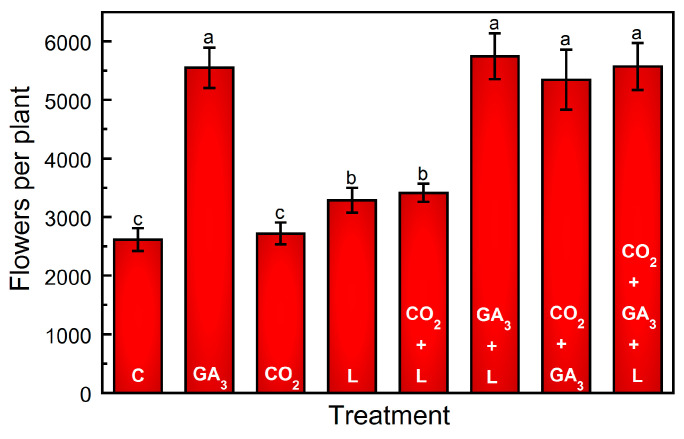
Influence of supplemental CO_2_, gibberellic acid (GA_3_), and light on flowering intensity of hop cv. ‘Centennial’. Treatments are control (C), exogenous application of 5 ppm GA_3_ to the apical bud basipetally through node 5 (GA_3_), supplemental intra-canopy light (L), supplemental intra-canopy CO_2_ (CO_2_), supplemental intra-canopy L + CO_2_, supplemental intra-canopy L + GA_3_, supplemental intra-canopy CO_2_ + GA_3_, and supplemental intra-canopy L + CO_2_ + GA_3_. Values are expressed as total cones per bine ± standard deviation of replicate plants *(n* = 9). Significant treatment differences are indicated using different letters in descending order (*p* < 0.01).

Compared to the control (C), significant flower set stimulation occurred in the GA_3_ treatment (*p* < 0.01) (Figure 1). Supplemental light (L) also resulted in an increase in flower set (*p* < 0.01) (Figure 1). However, the increase in flower set induced by L was not as substantial as that induced by GA_3_ (*p* < 0.01). Flower set increased by approximately 1000 flowers due to the within-canopy L treatment compared to the C treatment, whereas GA_3_ treatment resulted in approximately 2500 more cones per bine (Figure 1). Supplemental CO_2_ resulted in a similar flower set compared to C (*p* = 0.27). 

**Figure 2:**
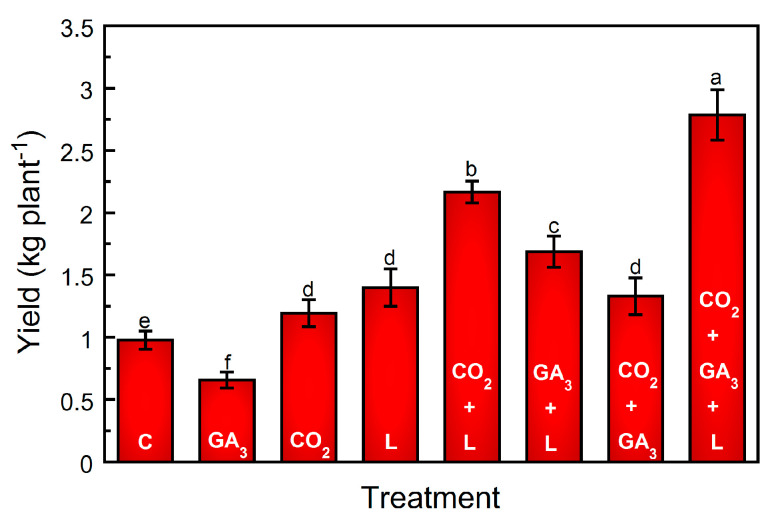
Influence of treatments on dry cone yield of cv. ‘Centennial’ (kg plant^−1^). Treatments are control (C), exogenous application of 5 ppm GA_3_ to the apical bud basipetally through node 5 (GA_3_), supplemental intra-canopy light (L), supplemental intra-canopy CO_2_ (CO_2_), supplemental intra-canopy L + CO_2_, supplemental intra-canopy L + GA_3_, supplemental intra-canopy CO_2_ + GA_3_, and supplemental intra-canopy L + CO_2_ + GA_3_. Values are expressed as means ± standard deviation of yield per treatment (*n* = 9 plants). Significant treatment differences are indicated using different letters in descending order (*p* < 0.01).

**Figure 3:**
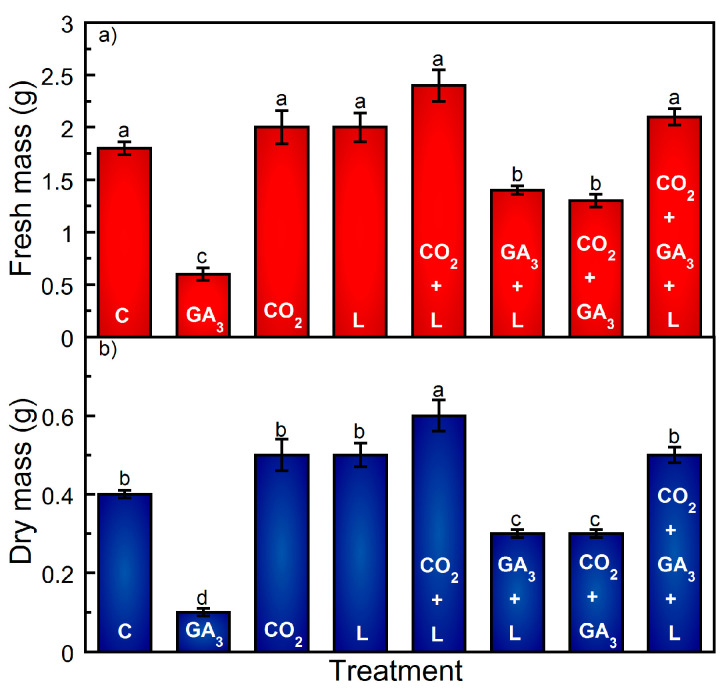
Influence of CO_2_, gibberellic acid (GA_3_), and light treatments on the mean fresh (**a**) and dry (**b**) cone mass of hop cv. ‘Centennial’. Treatments are control (C), exogenous application of 5 ppm GA_3_ to the apical bud basipetally through node 5 (GA_3_), supplemental intra-canopy light (L), supplemental intra-canopy CO_2_ (CO_2_), supplemental intra-canopy L + CO_2_, supplemental intra-canopy L + GA_3_, supplemental intra-canopy CO_2_ + GA_3_, and supplemental intra-canopy L + CO_2_ + GA_3_. Values are expressed as the mean of 150 g cone sub-samples for fresh and dry mass ± standard deviation of replicate plots per treatment (*n* = 3). Significant treatment differences are indicated using different letters in descending order (*p* < 0.01).

**Figure 4:**
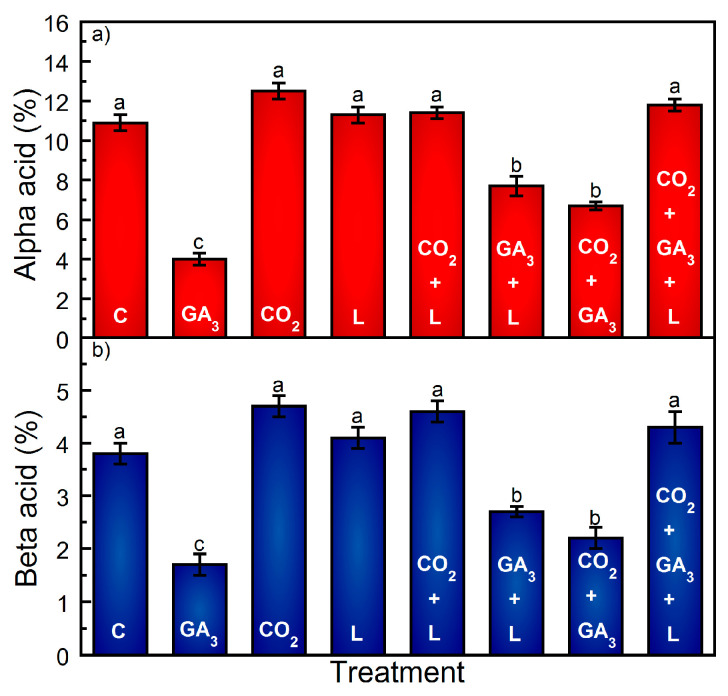
Observed alpha (**a**) and beta (**b**) bitter acid percentages and cone dry weight percentages of hop cv. ‘Centennial’ cones across treatments. Treatments are control (C), exogenous application of 5 ppm GA_3_ to the apical bud basipetally through node 5 (GA_3_), supplemental intra-canopy light (L), supplemental intra-canopy CO_2_ (CO_2_), supplemental intra-canopy L + CO_2_, supplemental intra-canopy L + GA_3_, supplemental intra-canopy CO_2_ + GA_3_, and supplemental intra-canopy L + CO_2_ + GA_3_. Observed means of 150 g cone sub-samples per treatment ± standard deviation. Significant alpha and beta-bitter acid treatment differences are indicated using different letters in descending order (*p* < 0.01).

Per plant, cone yield decreased by approximately 33% as a result of exogenous GA_3_ applied as an independent application (Figure 2), whereas L resulted in an approximate 43% increase (Figure 2). Supplemental CO_2_ increased cone yield by approximately 22% as compared to the C treatment, an estimate that is subject to the number of plants per hectare (Table 1). Relative to the 2023 Washington State Centennial cv. yield averages, the mean values for C ranged from −1.7 to +12.2% per hectare, the range in yield being a product of the variation in plants per hectare (Table 1). GA_3_ as an independent treatment resulted in a per hectare yield decrease, ranging from −27.5 to −41% (Table 1). Supplemental CO_2_ resulted in a modest yield increase (18.3%) compared to the 2023 Washington State yield averages (Table 1). Similarly, and depending on the number of plants per hectare, the addition of full spectrum light within the canopy increased the yield by approximately 34% to 47% (Table 1). GA_3_ resulted in a decrease in cone fresh and dry mass, whereas CO_2_ and L did not have an effect on either (Figure 3). Visually, bracts on cones in the independent GA_3_ treatment appeared loosely packed and airy compared to the appearance of cones that were not treated with GA_3_ (Supplemental Figure S1). The application of CO_2_ or L did not influence either fresh or dry cone mass as compared to the C treatment (Figure 3). There was a slightly significant increase in dry cone mass in the CO_2_ and L treatments (Figure 3). Analysis of α- and-β acids showed that the α- and β-acids were within the typical range for the cv. Centennial in C, CO_2_, and L. In comparison, GA_3_ as an independent treatment resulted in the largest decrease in α- and β-acid concentrations (Figure 4).

### 2.2. Combined Effects of CO_2_, GA_3_, and Light

Regardless of combining supplemental CO_2_ or L with the addition of GA_3_, cones per bine did not increase beyond those with GA_3_. Similarly, supplemental CO_2_ did not result in an increase in flower set when combined with L + GA_3_, beyond that of GA_3_ as a separate treatment (*p* = 0.37) (Figure 1). The addition of CO_2_ combined with GA_3_ resulted in a lower yield increase as compared to L + GA_3_ (36% versus 73%) (Figure 2). However, the cones in both treatments had less mass and were visually smaller than those produced in the C treatment (Figure 3; Supplemental Figure S1). In comparison, the combination of CO_2_ + L increased the yield by approximately 122% compared to the C treatment. The combination of CO_2_ and L resulted in a synergistic effect; the yield increased by approximately 122%. The combination of CO_2_ + GA_3_ or L + GA_3_ increased the yield; however, the average cone mass was lower and the cone morphology appeared airy (Figure 3; Supplemental Figure S1). 

GA_3_ application nearly doubled flower set when applied in combination with CO_2_ or L, and a significant decrease in cone mass ensued (*p* < 0.01) (Figure 3). Visually, the combination of CO_2_ or L with GA_3_ resulted in loosely packed bracts (Supplemental Figure S1). There was a slightly significant increase in dry cone mass in treatments L + CO_2_ and CO_2_ + GA_3_ + L (Figure 3). Compared to the C treatment, the combination of GA_3_ with L + CO_2_ resulted in the largest increase in yield and average mass per cone (Figure 1 and Figure 3). Interestingly, the morphology of the CO_2_ + GA_3_ + L-treated cones changed from cylindrical to more spherical, along with demonstrating a wider cone girth (Supplemental Figures S1 and S2). Additionally, visual observations indicated a substantial increase in lateral length (Supplemental Figures S3 and S4).

In this study, treatments L + CO_2_ and CO_2_ + GA_3_ + L had the largest impact on yield increases relative to the C treatment (Figure 2). The combination of CO_2_ + GA_3_ + L percent yield difference was nearly double that of typical Washington State field production (Table 1; Supplemental Figures S3–S5). The highest per plant yield increase occurred as a result of a combination of CO_2_, GA_3_, and L (*p* < 0.01) (Figure 2). The combination of all three supplemental resources resulted in an approximate 185% per plant yield increase (Figure 2). In addition, cones in the CO_2_ + GA_3_ + L treatment group appeared more dense with a wider girth relative to the C treatment (Supplemental Figures S1 and S2). The average dry mass per cone and the α- and β-acid concentrations were not impacted in the CO_2_ + GA_3_ + L treatment (Figure 3 and Figure 4; Supplemental Figure S6). 

Analysis of α- and β-acids showed that α- and β-acids were within the typical range for the cultivar Centennial in the L + CO_2_, and L + CO_2_ + GA_3_ treatment groups (Figure 4). Additionally, CO_2_ + GA_3_ and L + GA_3_ produced lower than typical α- and β-acid concentrations as compared to the C group and the normal range for the cultivar Centennial. However, the added CO_2_ and L resources in the case of CO_2_ + GA_3_ and L + GA_3_, although lower than in the C group, resulted in a slight increase in α- and β-acid concentrations as compared to GA_3_ (Figure 4).

## 3. Discussion

Detailed measurements from hop gardens can enhance models of how climatic factors control hop yield, alpha- and beta-acids, and cone development [15]. In this study, our focus was on investigating how supplemental CO_2_, GA_3_, and light treatments would influence hop quality and yield. Previous hop research consistently observed a significant increase in flower set from the application of exogenous GA_3_ [2,4,5,6,7,8]. Flower proliferation amounts, nonetheless, are sensitive to the exogenous GA_3_ rate-of-application, gradients in biological maturation, and photoinduction timing [2]. However, hops’ economic value is based on harvestable biomass rather than flower morphology and quantity. Nonetheless, in other flowering plant species, flower set and yield parameters are often strongly correlated, e.g., [16] if GA_3_-induced flower set increase, it also leads to an overall rise in harvestable cone biomass, which could be of considerable commercial horticultural interest. However, our results indicate that despite the proliferation of flower set in exogenous GA_3_ treatments, there was a decrease in cone mass, alpha- and beta-acids, and yield. Additionally, the rate of flower maturation decelerated relative to other study treatments. This observation suggests that the amount of carbohydrates produced was inadequate to support the increased flower set due to carbohydrate distribution to a larger flower sink. Other studies have also observed a deceleration of flower maturation rate and a lengthening of the cone development phase due to exogenous GA_3_ application in hops [3,8], which is a likely consequence of lower carbohydrate production when CO_2_ or light are limiting [1,3]. Hence, the use of GA_3_ for hop flower proliferation in regions with finite growing seasons could compromise flower development and negate any benefits of GA_3_-induced flower proliferation [8].

Treatments without a GA_3_ application produced typical cv. Centennial concentrations of α- and β-acid. Furthermore, cone mass and yield did not decrease in treatments without exogenous GA_3_ application. It is known that supplemental light increases hop flower set [11]. Our results illustrate an approximate 23% increase in flower set due to the addition of within-canopy full-spectrum supplemental light. The development of the most fructiferous hop laterals occurs within the crown near the juvenile-to-adult phase change, and supplementary light intensity within this area would promote photosynthesis and flower set, an area of the crown that might otherwise be light-limited due to light gradients along the hop vertical canopy depth profile [1,11]. In this study, the additional carbon assimilation generated from within-crown supplemental light could explain why cones matured and yield increased [2,8].

CO_2_ enrichment can increase the herbaceous flower yield by 10–50%, e.g., [16,17,18,19,20] and result in an increase in flower quality, e.g., [21,22]. Our CO_2_ enrichment results primarily affected the latter; hops exposed to CO_2_ enrichment had slightly higher α- and β-acids (*p =* 0.01). In comparison to supplemental light, CO_2_ enrichment resulted in less yield enhancement, approximately 22% versus approximately 43% for the light treatment. CO_2_ enrichment only benefits photosynthesis in the presence of light, and it is not uncommon for lower layers of a hop canopy to be light-limited due to light gradients that result in sub-optimal light conditions at lower canopy layers [1]. We also observed a hop yield increase from the combination of GA_3_ and CO_2_, but at the expense of a cone mass and α- and β-acid decrease. This result suggests that hop dry matter development is preferential to cone secondary metabolite synthesis, and α- and β-acids are not achieved without additional carbohydrate resources [3], a result equivalent to the competition for assimilates between vegetative and reproductive growth [23,24,25]. 

The combination of GA_3_ and light resulted in a larger yield increase compared to CO_2_ or GA_3_ alone. This illustrates that although exogenous GA_3_ increases flower set, it must be accompanied by additional carbohydrate-generating stimulants to attain extra cone dry matter [3,23,24,25]. Supplemental CO_2_ did not increase flower set; therefore, cone biomass gains were limited to an increase in cone mass and quality. Nonetheless, future atmospheric CO_2_ concentrations might mitigate temperature fluctuations that increase cone respiratory loss [26]. Concentrations of α- and β-acid are the accepted industry standard by which the quality of hop flowers are judged. Therefore, combining GA_3_ and light to increase yield is not of value if it decreases hop quality.

Typical of the combined effect of elevated CO_2_ and supplemental light, we observed a cone yield increase. Relative to leaf level photosynthetic responses to light and CO_2_, the yield response patterns we observed at the whole plant level agree with the hop leaf-level light and CO_2_ gradient response patterns reported by Bauerle [1]. Likewise, cone carbon assimilation responds to light gradients and the concentration of CO_2_ in the atmosphere [3]. Light increased hop flower set and in combination with elevated CO_2_, the extra carbon assimilation resulted in a synergistic effect; the additional cones matured and yield increased. The question thus arises as to whether the combination of all three (CO_2_ + GA_3_ + L) bolsters hop yield beyond the combination of two? In this study, the combination of CO_2_ + GA_3_ + L resulted in a significantly larger yield than any other treatment. The results suggest that substantial cone yield increases are the result of supplemental resources that permit carbon assimilation to proceed to a higher rate. Additionally, the application of CO_2_ + GA_3_ + L appears to lessen the hop yield gap, the difference between current hop yields, as compared to the upper limit of the plant’s genetic potential. 

Implementing supplemental light and CO_2_ in controlled environments involves significant economic costs, which can outweigh the benefits [27]. Metal-halide lamps, used for supplemental lighting in this study, have a high energy consumption, leading to increased electricity costs. Additionally, they generate significant heat, necessitating extra cooling systems. Despite these costs, the benefits can be substantial. Metal-halide lamps provide a broad spectrum of light that mimics natural sunlight, promoting plant growth and enhancing hop cone yield. We show that combined with CO_2_ enrichment, they can significantly boost hop productivity without compromising cone quality. While CO_2_ enrichment systems are also costly, the resulting yield improvements and quality enhancements can be economically beneficial in a niche market. Wet hops, freshly harvested and undried, are highly sought after for their unique flavors and aromas in craft brewing. The potential for off-season and year-round wet hop production can meet niche market demand, allowing growers to command premium prices. In summary, although the economic costs of implementing metal-halide lighting and CO_2_ enrichment are significant, the potential for premium pricing from off-season wet hop production and increased annual yields can make it economically viable. Careful planning, resource optimization, and detailed cost–benefit analysis are essential for ensuring the financial success of off-season hop production in controlled environments.

As far as the author knows, the maximum yield potential of hops is unknown. Roberts et al. [28] emphasize that breeding for increases in cones per bine and lateral-length could double cone yields. Roberts et al. [28], in an evaluation of 29 female hop cultivars, estimated that selecting and breeding for the number of cones per bine and yield per plant could result in a two-fold increase in yield, with more than 70% of the gain deriving from lateral length and cones per lateral. Studies have left the literature void of additional hop breeding techniques that could bolster genetic yield potential. Likewise, few studies in the literature focus on production techniques that lend themselves to increasing hop cone yield. In our study, cones per bine increased substantially due to the application of exogenous GA_3_, and CO_2_ + GA_3_ + L increased lateral length (e.g., Supplemental Figure S4). Moreover, although we did not measure lateral length, we witnessed a visually apparent increase in lateral length and cones per bine in the CO_2_ + GA_3_ + L treatment relative to Roberts et al. [28]. Variables that had the greatest influence on yield (Supplementary Figures S3–S5) [28] (Supplementary Figures S3–S5).

Crop performance studies that focus on increasing yield through improved horticultural production techniques have resulted in more than a 200% yield increase, e.g., [29,30] (pp. 180–202), yet the literature in this area of research remains lacking for hops. Similarly, the yield potential of new cultivars tested in plant breeding trials is probably greatly underestimated because of growth limitations resulting from insufficient resources. Currently, most hop research focuses on yield detriments, e.g., [15,31,32], and far fewer reports focus on genetic yield potential, particularly regarding cone yield per plant [26,33,34,35,36]. Thus, there is a strong need for experimentation that focuses on the aspects that enhance and maximize hop growth and yield. 

Haunold et al. [36] reported Oregon State yields of 3811 kg ha^−1^ at a plant density of 1912 plants per hectare, which equates to 4379 kg at a plant density of 2197 per hectare. Similar yields have been reported for light-intensive locations such as Australia and Washington State [4,37,38]. Recently, reports by Nevadba et al. [35] suggest that cone yield values of 6000 kg per hectare are feasible, a quantity unearthed by the hop yield world record set in 1891 [39]. Selecting for increased lateral length and/or number of cones per vine is the best path forward for obtaining high-yielding hop cultivars [28,33]. Our study indicates that in addition to exogenous growth hormone effects on cones per plant, investigating the hormone effects on hop lateral length is warranted as well (e.g., Supplemental Figure S4).

## 4. Conclusions

The Centennial cv. yield averages were typical of the field-grown USDA cv. Centennial selection [40]. Overall, the combination of CO_2_ + GA_3_ + L showed the most significant increase in yield, while maintaining brewing quality. As compared to the highest yield on record, our results fell slightly short [39]. Nonetheless, the combination of multiple supplemental resources significantly bolstered hop yield without a deleterious effect on hop quality attributes. Additional research is needed to close the hop yield gap between current hop yields and the achievement of the plant’s genetic potential.

## 5. Materials and Methods

### 5.1. Environmental Conditions

Primary measurements occurred during 2019–2020 at the Horticulture Center of Colorado State University, Fort Collins, CO. A *Humulus lupulus* L. common garden was established within a 12.2 by 24.4 m active and passive ventilation greenhouse. When temperatures were close to the setpoint, roof vents were open. Cool replacement air entered windward via a 10 m by 1.7 m open end wall that was fitted with a 15.24 cm thick evaporative pad system. If temperatures exceeded 2 °C above the 24 °C setpoint, four unidirectional 0.92 m 1 hp exhaust fans forced air outward through the leeward end wall. The bottom of the fans were located 1.25 m above the floor to ensure the replacement air flowed over and through the plant canopy. Set point conditions were controlled as follows: air temperature 24 °C during photoperiod and 18 °C during the dark with a 45 min temperature step change between the two, relative humidity (RH; %) of 50% with supplemental humidity provided via air humidification from the evaporative pad (Wadsworth Control Systems, Arvada, CO, USA). Per row, overhead LED linear lighting, mounted to the purlin at the top of each trellis, provided photosynthetically active radiation (PAR) directly above the canopy (Philips lighting N.V., Amsterdam, The Netherlands). Within each row, two intra-canopy LEDs were suspended at 2.5 and 3.25 m above the Earth’s surface. The top of the canopy LED supplemental PAR ranged from 500 to 600 μmol m^−2^ s^−1^ and intra-canopy ranged from 150 to 300 μmol m^−2^ s^−1^. Over the experimental period, average daytime temperatures were 24.6 °C, vapor pressure deficit (VPD) was 1.6 kPa, and ambient PAR above the canopy lighting was generally 800–1200 µmol m^−2^ s^−1^ at solar noon.

### 5.2. Plant Material

The public cultivar ‘Centennial’ was used due to its wide availability and substantial use in the brewing industry. Propagation occurred via tissue culture (Summit Plant Labs Inc., Fort Collins, CO, USA). Ninety plants were grown with plants spaced 1.2 m within rows and rows were spaced 2 m apart. Initially, all pots were watered to saturation and permitted to drain for 18 h. Numerous times daily, an automated irrigation system supplied nutrients via the irrigation delivered through pressure-compensating drip emitters, as described in Bauerle [14]. Day length extension (19 h) occurred via the LED illumination. One bine per container was initially trained on a 1.3 m bamboo stick and was periodically repositioned to coil juvenile nodes around the container. The procedure permitted the location of adult node 25 at a uniform height of 0.9 m, with approximately 4 m of initial juvenile bine coiled around the container. From a height of 0.9 m, the bine was trained through vertical net trellis using the internode compactness and increased node density bending technique described by Bauerle [14]. Once 35 visual nodes developed, plant leaves and side laterals were removed from the base of the plant up through node 24 to eliminate non reproductive tissue and to increase intra-canopy light and air penetration. Spotted mites, whitefly, and thrips were controlled by the beneficial mite *Amblyseius swirskii* (Amblyforce^TM^ S, Beneficial Insectary, Inc. Redding, CA, USA). 

### 5.3. Treatments

A randomized complete block design (RCBD) was established with three plants per plot, replicated three times. Treatments were (1) ambient conditions as a control (C), (2) exogenous application of 5 ppm GA_3_ to the apical bud basipetally through node 20 as described in Bauerle [2] (GA_3_), (3) additional intra-canopy light (L), (4) supplemental intra-canopy CO_2_ (CO_2_), (5) supplemental intra-canopy L + CO_2_, (6) supplemental intra-canopy L + GA_3_, (7) supplemental intra-canopy CO_2_ + GA_3_, and (8) supplemental intra-canopy L + CO_2_ + GA_3_. Treatments and photoperiod induction commenced once node 25 became visible to the naked eye. At that time, the photoperiod was reduced from 19 to 15 h, and treatment applications occurred that evening. Treatment L consisted of 350 W Philips metal-halide lights (Philips lighting N.V., Amsterdam, The Netherlands) suspended horizontally between the rows at 2 m above the Earth’s surface. The full spectrum metal-halide lights provided treatment L with ~500 μmol m^−2^ s^−1^ of additional PAR at the canopy periphery. The enriched CO_2_ treatment employed one target CO_2_ level, 750 µmol mol^–1^. As an *a priori* CO_2_ cross contamination precaution, a vertical plastic CO_2_ diffusion barrier separated the CO_2_ treatment. The barrier was constructed parallel to the unidirectional flow of forced-air replacement and acted to avoid lateral gas diffusion between CO_2_-enriched and non-enriched treatments. In addition, buffer plants were placed between CO_2_-enriched and non-enriched treatments to increase the horizontal distance to at least 6 m between enriched and non-enriched treatments. CO_2_-free air concentration enrichment (FACE) was deployed within individual hop crowns using laser-drilled flexible tubing specifically designed for CO_2_ fumigation (CO_2_ Rain^®^ System, Sunlight Supply, Inc., Vancouver, WA, USA). The tubing was interwoven vertically along the bines beginning at 1.5 m from the floor surface and terminating at 3 m, which permitted the delivery of liquefied food-grade CO_2_ emitted at pressure within each crown and immediately adjacent individual bines. The tubing’s outside and inside thicknesses were 4.76 mm and 3.18 mm, and CO_2_ emission holes were 0.5 mm in diameter spaced 15 cm apart (Titan Controls^®^ CO_2_ Rain^®^ System, Sunlight Supply, Inc., Vancouver, WA, USA). The tubes were attached to a manifold at the tank emission source where the CO_2_ volume flow rate into the distribution manifold was 0.25 m^3^/h ± 2.5% regulated at 0–300 kPa CO_2_ working pressure (Titan Controls^®^ Manatee CO_2_ Regulator, Sunlight Supply, Inc., Vancouver, WA, USA). The within-canopy CO_2_ concentration enrichment status was measured and adjusted at 10 s intervals using altitude-corrected CO_2_ controllers (model RAD 0501, CO_2_Meter Inc., Ormond Beach, FL, USA). Periodic monitoring of CO_2_ at the surface of each bine showed CO_2_ concentrations ranging from 623 to 846 ppm (model RAD 0501, CO_2_Meter Inc., Ormond Beach, FL, USA). Due to continuous active or passive ventilation, CO_2_ concentrations 0.75 m perpendicular the CO_2_-enriched hop crowns decreased to approximately 500 µmol mol^–1^. CO_2_ fumigation was provided from 8:00 to 18:00, and criteria established by Nagy et al. [41] was used as a benchmark for the target CO_2_ concentration at the bine surface. CO_2_ was maintained within 20% of 750 µmol mol^–1^ for over 80% of the time. Thus, our elevated CO_2_ set point of 750 µmol mol^–1^ corresponded to ±150 µmol mol^–1^ CO_2_ (600–900 µmol mol^–1^). In addition, the non-enriched treatments were periodically monitored to ensure they did not significantly deviate from near-ambient CO_2_ concentrations. 

### 5.4. Cone Mass, Quantity, and Yield

Eight weeks after treatment initiation, strobili were manually harvested. Per replicate plot, the mass of 150 random cones was averaged, and fifty fresh hop cones were randomly selected per replicate (50 cones per three plant replicate plot). Fresh cone mass was immediately measured at approximately 75% moisture. Next, the number of hop inflorescences per plant was tallied. Immediately thereafter, the cones were dried for approximately 7 h at 43 °C in a forced air dryer until reaching the industry dry cone storage value of 8–9% moisture. The 150 randomly selected cones per replicate plot were kept separate for calculations of average cone fresh and dry mass based on the following formulas:

(1) Average cone fresh mass=Mass of 150 fresh hop cones/150

(2) Average cone dry mass=Mass of 150 dry hop cones/150

Immediately after drying, dry cone mass was recorded.

### 5.5. Cone Moisture Content

Approximately 10 g of fresh hop cones was dried at 70 °C until weights stabilized. The sample weight was measured before and after drying, and the cone moisture content was calculated based on the following formula:

(3) Moisture content=Mass before −Mass after/Mass before ×100%

where mass is expressed before and after drying. 

### 5.6. Cone Alpha- and Beta-Acid Content

Cone alpha- and beta-acid contents were determined using the spectrophotometric technique on sub-samples of cones, 150 g from each plot [42]. Samples were ground to a fine powder and a homogenized sample was extracted from the lot of dried raw hops; a total of 2.5 g of dried hop powder was weighed to the nearest mg and placed in a 100 mL beaker with 50.0 mL of methanol. The aliquot was stirred for 30 min at room temperature and the extract was then force-filtered via centrifuge to remove particulate matter. A 50 μL aliquot of the filtrate was placed in a 25 mL volumetric flask and the flask was then filled with methanolic NaOH (0.5 mL of 6 M NaOH in 250 mL of methanol). An aliquot of this solution was placed in a 1 cm quartz cell and its absorbance values were obtained at the wavelengths of 275, 325, and 355 nm against a blank of 50 μL of methanol in 25 mL of methanolic NaOH (Hach 6000 spectrophotometer; Loveland, CO, USA). Cone alpha- and beta-acid absorbance was calculated based on the following relationship between absorbance, concentration, and path length:

(4) A=ε×c×l

where *A* is absorbance, ε is the molar absorptivity coefficient, *c* is the concentration of the compound in solution, and *l* is the path length of the cuvette.

### 5.7. Statistical Analyses

Plant yield, number of cones per plant, alpha- and beta-acid concentrations, and fresh and dry cone mass were analyzed using two-way ANOVA (*p* < 0.01), which included fixed effects for the main treatment factor (CO_2_), the nested factors (L, GA_3_, L + GA_3_), and their interactions, as well as a random effect to account for the blocking structure. A preplanned Bonferroni post hoc correction was applied for multiple pairwise comparisons (SPSS, Version 28, IBM Analytics, www.ibm.com/analytics/ 15 June 2024, Armonk, NY USA).

## Figures and Tables

**Table 1:** The percent difference (%) of the average dry cone yield per ha^−1^ for USDA accession number 21507 (hop cv. ‘Centennial’; 1900 kg per ha^−1^) versus this study at two planting densities, 2197 and 1912 plants per ha^−1^. Treatments are control (C), exogenous application of 5 ppm GA_3_ to the apical bud basipetally through node 5 (GA_3_), supplemental intra-canopy light (L), supplemental intra-canopy CO_2_ (CO_2_), supplemental intra-canopy L + CO_2_, supplemental intra-canopy L + GA_3_, supplemental intra-canopy CO_2_ + GA_3_, and supplemental intra-canopy L + CO_2_ + GA_3_. Note, observed versus field-grown yield comparisons represent one crop cycle per annum.

Treatment	% 2197 (plants/ha)	% 1912 (plants/ha)
C	+12.2	−1.7
GA_3_	−27.5	−41
CO_2_	+31.9	+18.3
L	+47.2	+33.9
L + CO_2_	+85.9	+74.2
L + GA_3_	+64.4	+51.8
CO_2_ + GA_3_	+42.3	+28.9
CO_2_ + GA_3_ + L	+105.2	+94.8

## Data Availability

The data presented in this study are available on request from the corresponding author.

## Supplementary Materials

The following supporting information can be downloaded at: https://www.mdpi.com/article/10.3390/plants13121670/s1, Figure S1: Representative cone visual morphology for a cone from each treatment.; Figure S2: A lateral of a hop bine treated with CO_2_, gibberellic acid, and light located at node 70 of the bine; Figure S3: A lateral of a hop bine treated with CO_2_, gibberellic acid, and light located at node 60 of the bine; Figure S4: A lateral of a hop bine treated with CO_2_, gibberellic acid, and light located at node 30 of the bine; Figure S5: A side view of a hop crown segment treated with CO_2_, gibberellic acid, and light.; Figure S6: A quarter sectioned hop cone at maturity from the CO_2_, gibberellic acid, and light treatment.
