# Supplementary material for: Separate and Combined Effects of Supplemental CO2, Gibberellic Acid, and Light on Hop Quality and Yield
Source: Plants (Basel). 2024 Jun 16;13(12):1670. doi: 10.3390/plants13121670 (PMC11207437; doi:10.3390/plants13121670)
Supplement: Supplementary file 1 [file plants-13-01670-s001.zip › plants-3027912-supplementary.pdf]

Supplemental information

Separate and Combined Effects of Supplemental CO_2, Gibberellic Acid, and Light on Hop Quality and Yield

William L. Bauerle

Department of Horticulture and Landscape Architecture, Colorado State University,
Fort Collins, CO 80523, USA; bauerle@colostate.edu

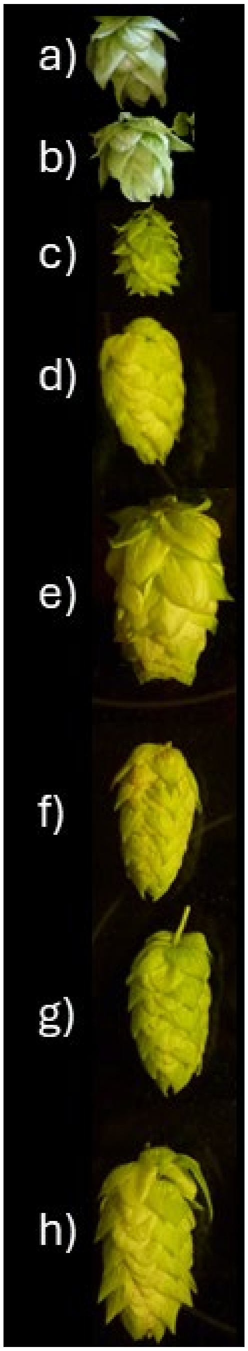

Supplemental Figure S1. Representative cone visual morphology for a cone from each treatment. Treatments associated with each cone are a) GA_3, b) GA_3 + CO_2, c) GA_3 + light, d) control, e) GA_3 + CO_2 + L, f) CO_2, g) light, and h) + CO_2 + light. Note, treatments GA_3 and GA_3 + CO_2 also resulted in pale green cones relative to the other treatments.

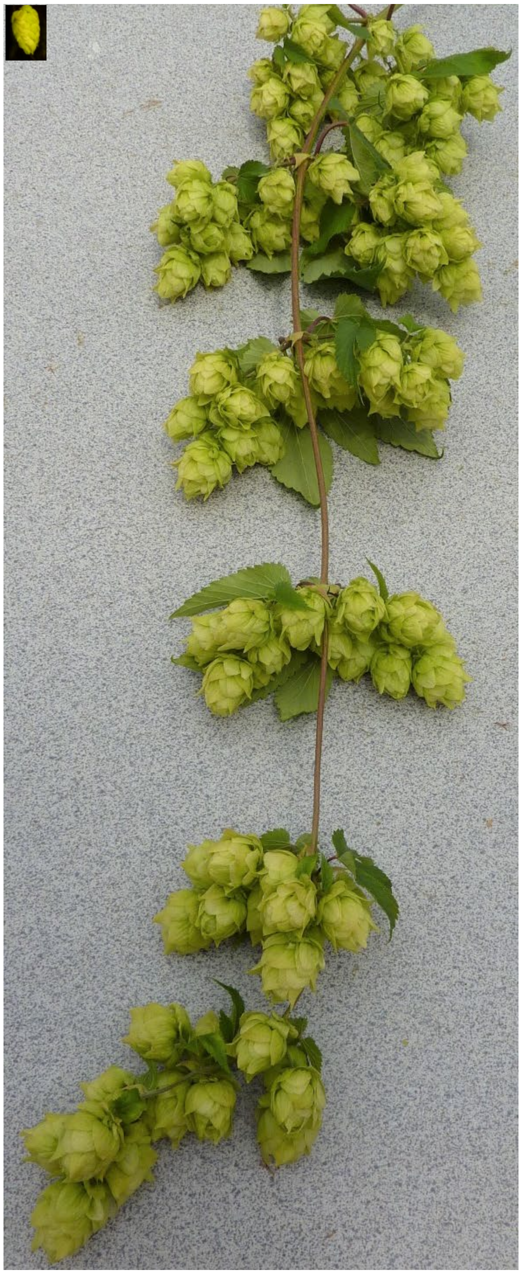

Supplemental Figure S2. A lateral of a hop bine treated with CO_2, gibberellic acid, and light located at node 70 of the bine (proximate to top of the hop crown). Relative to the numerous cones per node, only two leaves emerged per node. The picture also illustrates the prolific and dense nature of cone development at each node. For comparative purposes, a control cone is inset in the top left of the picture.

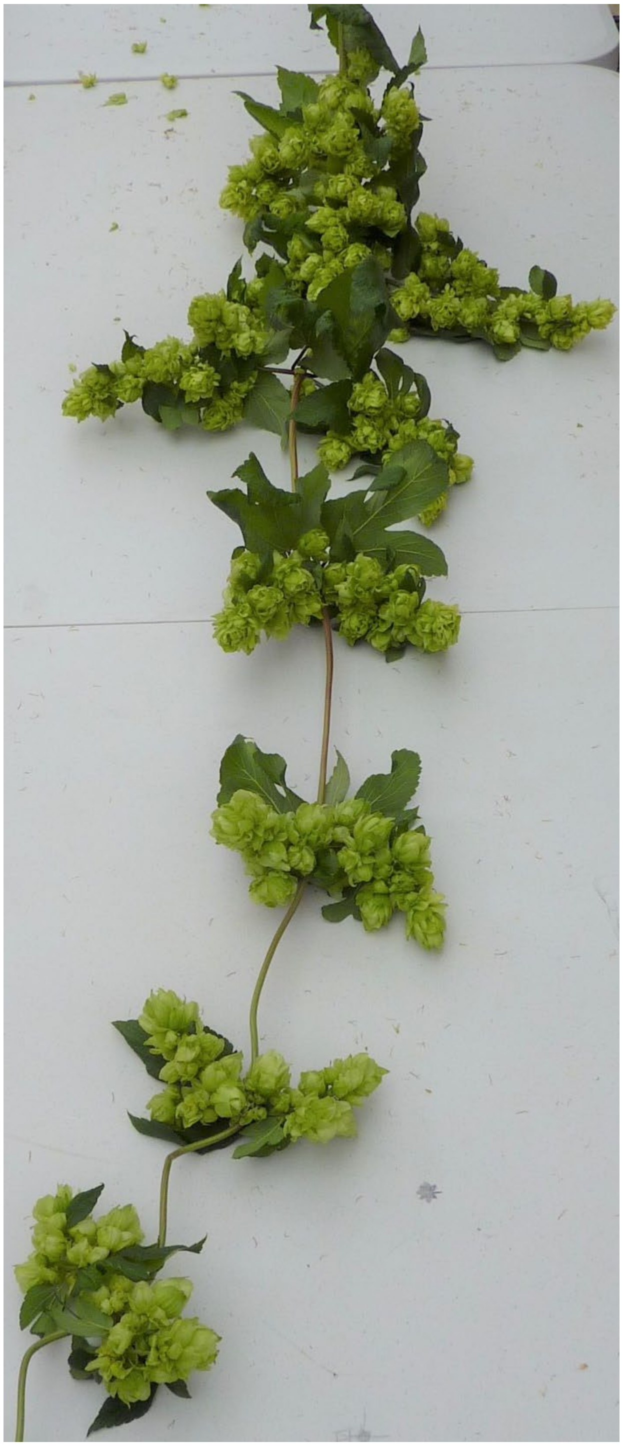

Supplemental Figure S3. A lateral of a hop bine treated with CO_2, gibberellic acid, and light located at node 60 of the bine (within the top 1/3 of the hop crown). The picture illustrates prolific and dense cone development within the CO_2, gibberellic acid, and light treatment as well as compact cone clustering at each node.

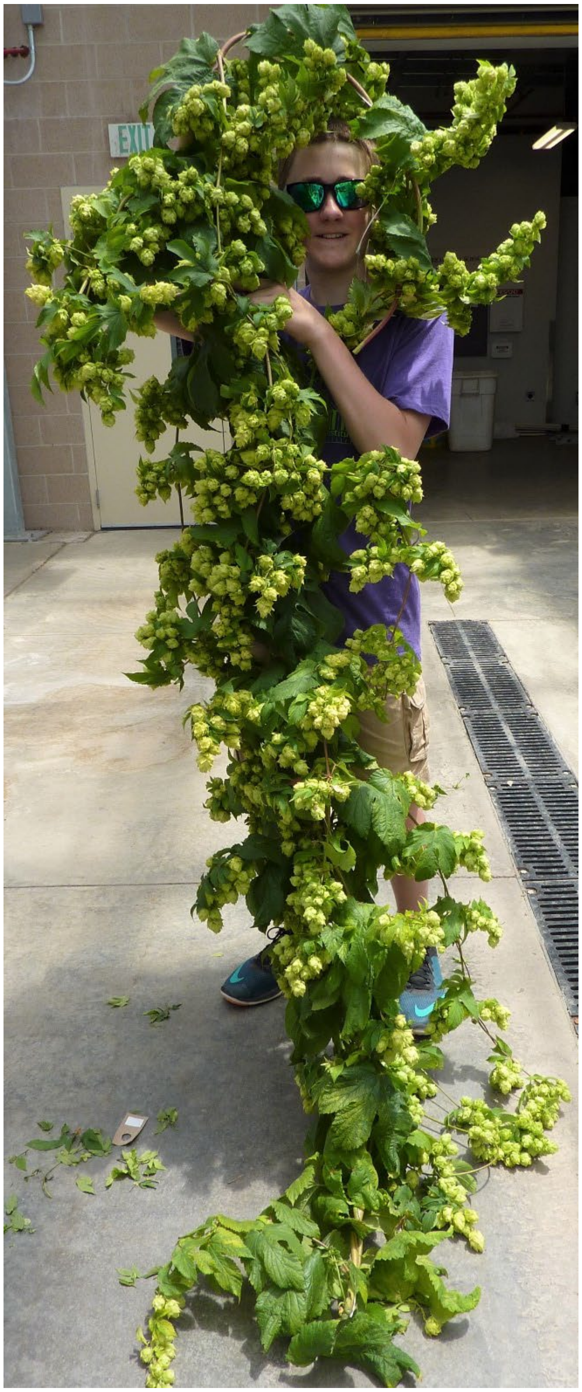

Supplemental Figure S4. A lateral of a hop bine treated with CO_2, gibberellic acid, and light located at node 30 of the bine (proximate to the most fructiferous portion of the hop crown). The person holding the lateral is 1.65 m tall, a reference for lateral length and cone quantity at the most fructiferous portion of the hop bine.

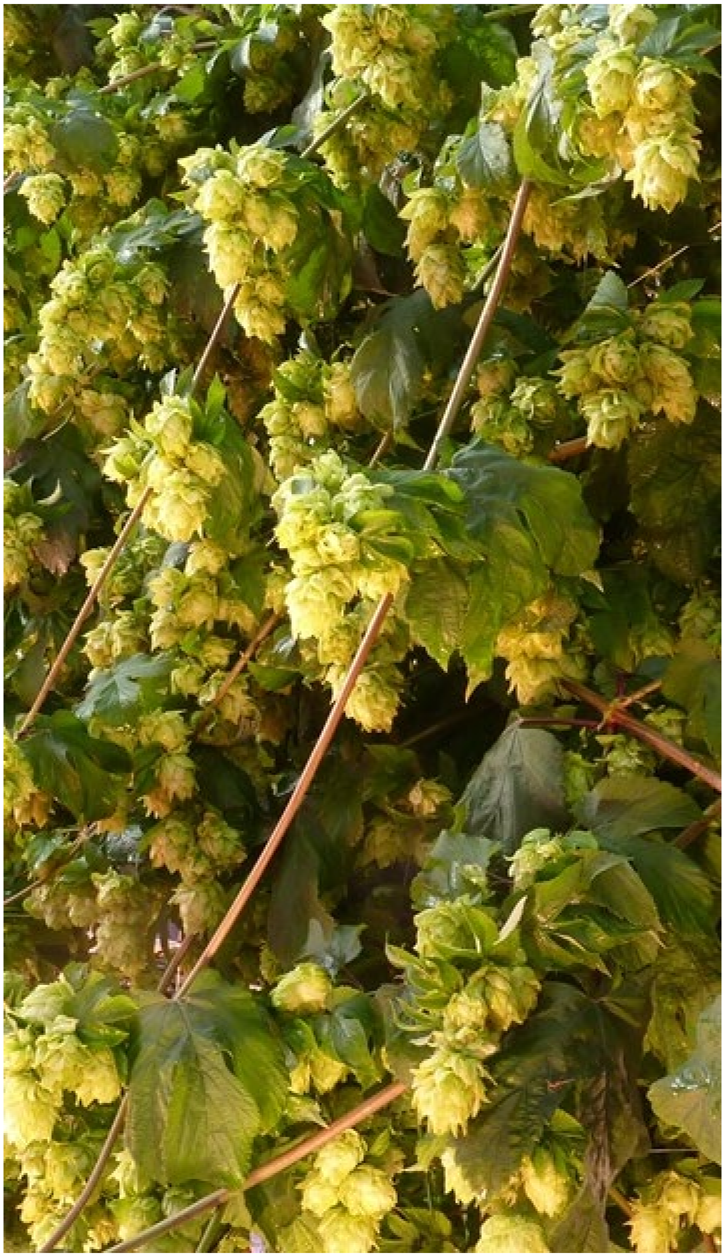

Supplemental Figure S5. A side view of a hop crown treated with CO_2, gibberellic acid, and light. Prolific cone development occurred on cascading laterals. In addition, the two leaves that developed per node tended to bend inwards toward the center of the crown due to dense cone clustering, which allowed cones to protrude to the canopy periphery.

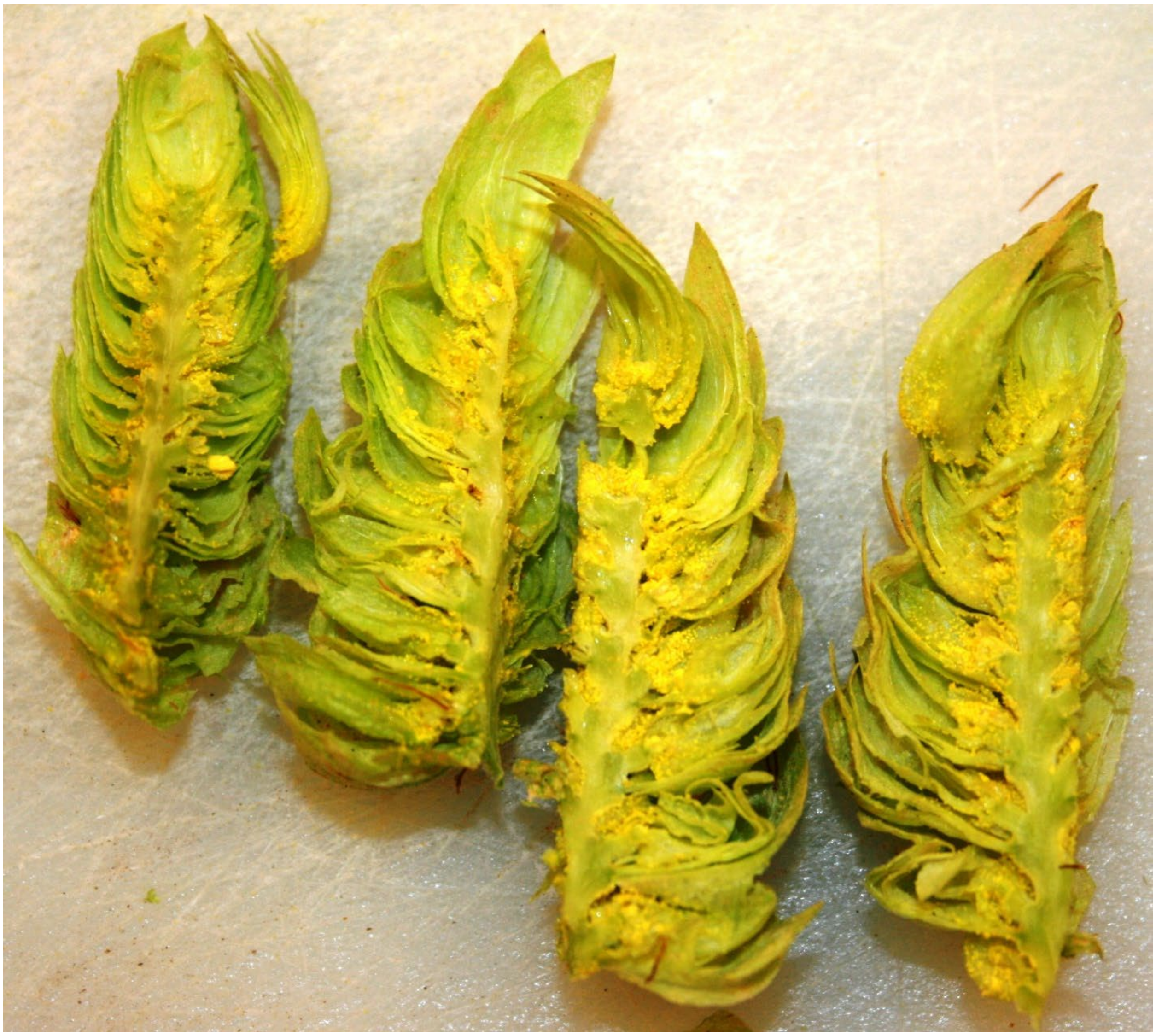

Supplemental Figure S6. A quarter sectioned hop cone at maturity from the CO_2, gibberellic acid, and light treatment. The picture illustrates the quantity of clustered lupulin glands near the base of the bracts and glistening from the hop cone oil content.
